# The Effects of Lateral Meniscus Posterior Root Tear and its Repair on Knee Stability of Internal Rotation and Forward Shift: A Biomechanical Kinematics Cadaver Study

**DOI:** 10.3389/fbioe.2021.792894

**Published:** 2022-01-19

**Authors:** Yan-Song Qi, Hu-Ri-Cha Bao, Li-Yuan Tao, Pei-Liang Gu, Chao-Le-Men Kong, Jun-Chen Wang, Yong-Sheng Xu

**Affiliations:** ^1^ Department of Orthopedics, Inner Mongolia People’s Hospital, Hohhot, China; ^2^ Research Center of Clinical Epidemiology, Peking University Third Hospital, Beijing, China; ^3^ Department of Histology and Embryology, School of Basic Medical Science, Peking University Health Science Center, Beijing, China; ^4^ School of Mechanical Engineering and Automation, Beihang University, Beijing, China

**Keywords:** lateral meniscus posterior root tear, knee stability, kinematics, internal rotation instability, anterior shift instability

## Abstract

**Objective:** Lateral meniscal posterior root (LMPR) is an important stabilizer for knee joint, providing the stability during tibia forward shifting and internal rotating. It is still controversial that whether the LMPR tear (LMPRT) should be repaired together with ACL reconstruction. This study aims to investigate the effects of LMPR on knee stability with intact ACL.

**Methods:** Eight cadaver knees were used and performed the biomechanical kinematics tests in orders of: Group A: the LMPR was intact; Group B: the LMPR was cut off from its tibial end; Group C: the LMPRT has been repaired. 1) An internal rotation moment (5 Nm) was given to the tibia, then the internal rotation angle of the tibia was measured; 2) An forward shifting force (134 N) was given to the tibia, then the anterior displacement of the tibia was measured; 3) An internal rotation moment (5 Nm) and a valgus moment (10 Nm) were given to the tibia, then the internal rotation angle and the anterior displacement was measured. The stability was inferred from smaller rotation angle and displacement, and all of the angles and displacements were measured at knee flexion of 0°, 30°, 60° and 90°, respectively.

**Results:** Comparing to Group A, the internal rotation angle in Group B was increased significantly at knee flexion of 30° (*p* = 0.025), 60° (*p* = 0.041), 90° (*p* = 0.002); the anterior tibia displacement in Group B was increased significantly at knee flexion of 30° (*p* = 0.015), 60° (*p* = 0.024); at knee valgus, the internal rotation angle was also increased significantly at knee flexion of 60° (*p* = 0.011), 90° (*p* = 0.037). Comparing to Group B, the internal rotation angle in Group C was decreased significantly at knee flexion of 30° (*p* = 0.030), 60° (*p* = 0.019), 90° (*p* = 0.021); the anterior displacement in Group C was decreased significantly at knee flexion of 30° (*p* = 0.042), 60° (*p* = 0.037); at valgus, the internal rotation angle was also decreased significantly at knee flexion of 60° (*p* = 0.013), 90° (*p* = 0.045). Comparing to Group A, only the internal rotation angle (*p* = 0.047) and anterior displacement (*p* = 0.033) in Group C were increased at knee flexion of 30°.

**Conclusion:** In simulated knee with intact ACL, LMPRT can still lead to the notable internal rotational instability at knee flexion from 30° to 90°, as well as the anterior shift instability at knee flexion from 30° to 60°. LMPRT repair help to improve the internal rotation stability at 30° and restore it at 60° to 90°, and improve the anterior shift stability at 30° and restore it at 60°.

## Introduction

Meniscal posterior root (MPR) refers to the attachment site of meniscus posterior horn to the intercondylar region on tibial plateau. (Wang et al., 2021). Meniscal posterior root tear (MPRT) is defined as a tear or avulsion injury within 1 cm of the MPR tibia attachment point. (Ahn et al., 2009). MPR is essential for maintaining the normal alignment and physiological function of the knee. Lateral MPRT (LMPRT) is associated with sports injury and trauma. (Brody et al., 2006).

MPR plays an extremely important role in transforming the load and maintaining knee stability. (Levy et al., 1982; Shybut et al., 2015). LMPRT leads to meniscus extrusion and kinematic changes during the joint motion, because of lack of the tibial anchor, which is equal to an invalid meniscus. It has been reported that LMPRT can affect the distribution of knee pressure load. (Ode et al., 2012; Schillhammer et al., 2012). What’ more, LMPRT also lead to the instability. It has been known that the lateral MPR is another essential stabilizer for knee joint (the second), following the anterior cruciate ligament (ACL). (Levy et al., 1982; Shybut et al., 2015). As the knee flexion angle increasing, the lateral MPR will become the primary stabilizer, providing the main stability of the internal rotation. (Pache et al., 2018). An intact lateral MPR can prevent the tibia over-forward shift and internal rotation during motions and sports injury.

LMPRT is commonly concomitant with ACL rupture. However, it is still controversial that whether and why the LMPRT should be repaired, together with ACL reconstruction. Excessive valgus force is the main injury mechanism for ACL rupture and LMPRT. LMPR will sustain much more stress from the lateral condyle when knee suffers from valgus force, making it contribute more to the joint stability, especially during internal rotating and forward shifting. At present, studies focused on how LMPRT affects knee stability is relatively limited, (Allaire et al., 2008), although many studies have focused on the load changes, (Schillhammer et al., 2012), or Osteoarthritis. (Ahn et al., 2010). There are only several case reports and clinical follow-up study of the LMPRT repair, (Ahn et al., 2010), lacking of the biomechanical effects of LMPRT on knee stability. We designed a cadaver study to investigate the effects of LMPRT on knee stability.

## Materials and Methods

### Cadavers and Specimens

Eight frozen knee joints donated from four male and four female cadavers were used in this study. The averaged death age was 53.25 ± 5.70 (range: 40–65) years old. The X-ray, MRI, and CT were perform before the experiment in order to exclude: 1) knee fracture and lower-extremity fracture; 2) meniscus injury; 3) ligaments injury; 4) severe osteoarthritis (Outerbridge Ⅲ-Ⅳ level); 5) knee surgery history. The experimental protocols and procedures have been approved by the Ethics Committee in our hospital. The cadaveric knee specimens involved in this study were provided by the Department of Histology and Embryology, School of Basic Medical Science, Peking University Health Science Center.

The tissues beyond 10 cm of joint space proximally and distally were removed, as well as the skin, subcutaneous tissues, muscles, deep fascia tissues were excised. The collateral ligaments, medical and lateral meniscus, and both cruciate ligaments were retained. The specimens were stored at −20°C, and thawed naturally at room temperature for 24 h before the experiment.

### Experimental Apparatus

The experiment test platform was set included the digital angle measuring instrument (AICE DXL360S, China) (Supplementary Figure S1A), a self-made corpse knee fixture (Supplementary Figure S1B), a robotic arm (Japan) (Supplementary Figure S1D), and a 6-demesional freedom force sensors (SRI M3705B, Japan) (Supplementary Figure S1E). The arthroscopic equipment and surgical instruments (Smith and Smith, America) were used.

### Biomechanical Kinematics Test

The knee joint was fixed to the experimental platform with Kirschner wires and clamps (Supplementary Figure S2A). The biomechanical kinematics tests were performed in the orders of the following: 1) Group A: the lateral MPR was intact (*n* = 8); 2) Group B: the lateral MPR was cut off from its tibial end (*n* = 8); 3) Group C: the LMPRT has been repaired (*n* = 8).

Because lateral MPR functions as the stabilizer on preventing the internal rotation and forward shift, we have detected the effects of LMPRT on these motion modes. Procedures of detecting the biomechanical kinematics on knee stability were listed as the following (Supplementary Figures S2B–D). 1) The internal rotation stability: an internal rotation moment of 5 Nm was given to the tibia (Supplementary Figure S2B), then the internal rotation angle of the tibia was measured at knee flexion of 0°, 30°, 60° and 90°, respectively. (Frank et al., 2017). 2) The anterior shift stability: a forward shifting force of 134 N was given to the tibia (Supplementary Figure S2C), then the anterior displacement of the tibia was measured at knee flexion of 0°, 30°, 60° and 90°, respectively. (Frank et al., 2017). 3) The internal rotation stability and anterior shift stability at knee valgus: an internal rotation moment of 5 Nm and a valgus moment with 10 Nm were given to the tibia (Supplementary Figure S2D), then the internal rotation angle as well as the anterior displacement of the tibia was measured at knee flexion of 0°, 30°, 60° and 90°, respectively. (Frank et al., 2017). Each procedure was repeated twice and averaged at each flexion degree.

**FIGURE 1:**
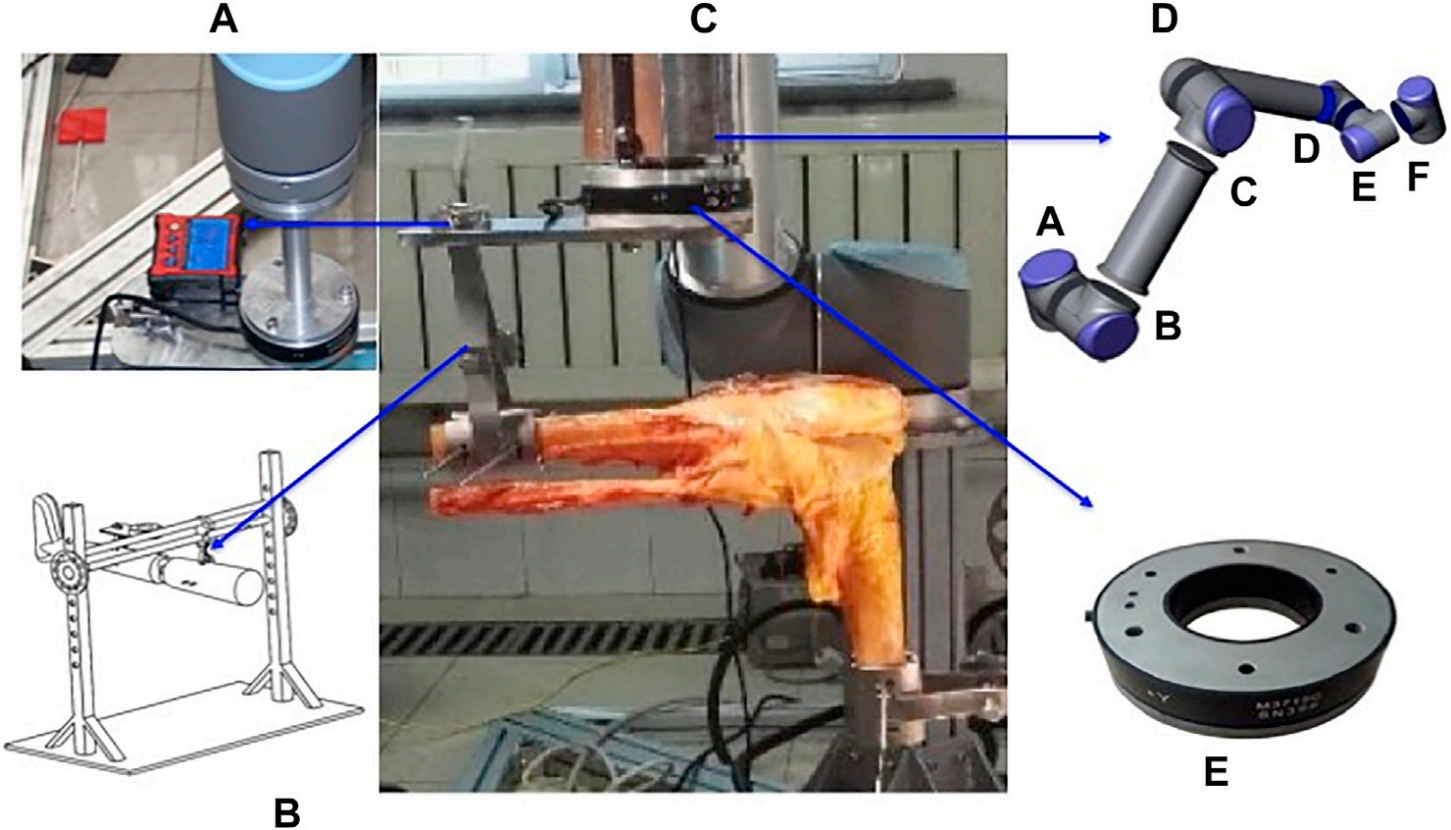
The biomechanical kinematics experiment test platform. Figure 1A, the digital angle measuring instrument; Figure 1B, a self-made corpse knee fixture; Figure 1D, the UR10 robotic arm; Figure 1E, 6-demesional freedom force sensors.

**FIGURE 2:**
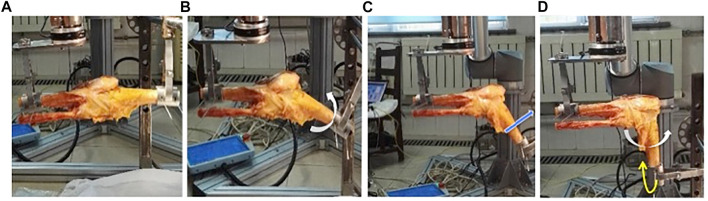
Biomechanical kinematics parameters and procedures. Figure 2A, at the beginning, the knee was fixed with Kirschner wires and clamps at 0°; Figure 2B, during the internal rotation stability test, an internal rotation load of 5 Nm (while arrow) was given to tibia, this figure shows the internal rotation angle was detected at 30°; Figure 2C, during the anterior shift stability test, a forward shifting load of 134 N (blue arrow) was given to the tibia, and this figure shows the anterior displacement was measured at 60; Figure 2D, during stability test knee valgus, an internal rotation load of 5 Nm (while arrow) and a valgus stress with 10 Nm (yellow arrow) were given to the tibia, this figure shows the internal rotation angle and anterior displacement were detected at 90.

### LMPRT Repair

The arthroscopy suture of LMPRT was performed. The tibia locator of ACL reconstruction was used to locate the MPR insertion site, and then a tibial tunnel with the diameter of 4.5 mm was drilled. The MPR was sutured with a No. 2 Ultrabraid wire, and which was pulled out of the tibial tunnel by a PDS guide wire. After adjusting for an appropriate meniscus tension (LMPR should not lead to incarceration during the flexion-extension and should be tested as stable by the arthroscopic probe), those sutures were knotted through a button plate, which was fixed on the tibia surface.

### Statistical Analysis

Measurement data were expressed as mean ± SD. Comparisons of the continuous data were processed by the one-way *ANOVA*, and the pair-wise comparisons were analyzed by *LSD* method. The level of significance was set at 0.05. All of the statistical analyses were performed using IBM SPSS 20.0 (SPSS Inc., 2009; Chicago, IL, United States).

## Results

### The Internal Rotation Stability

At knee flexion of 0°, there was no statistical difference of the internal rotation angle between the Group A, B and C (Supplementary Table S1). At knee flexion of 30°, 60° and 90°, the internal rotation angle in Group B was increased significantly comparing to Group A, and the internal rotation angle in Group C was decreased significantly comparing to Group B (Supplementary Table S1). At knee flexion of 60° and 90°, Group C and A had the comparable internal rotation angle, while at knee flexion of 30°, the internal rotation angle in Group C was still larger than that in Group A (Supplementary Table S1).

### The Anterior Shift Stability

At knee flexion of 0° and 90°, there was no significant difference of the anterior tibial displacement distance between the Group A, B and C (Supplementary Table S2). At knee flexion of 30° and 60°, the tibial anterior displacement was increased in Group B compared with Group A, and which were decreased significantly in Group C compared with Group B (Supplementary Table S2). At knee flexion of 30°, the tibial anterior displacement in Group C was still larger than that in Group A, while at knee flexion of 30°, the tibial anterior displacement in Group A and C was comparable (Supplementary Table S2).

### The Internal Rotation Stability and Anterior Shift Stability at Knee Valgus

The internal rotation angle between Group A, B and C had no significant difference at knee flexion of 0° and 30° (Supplementary Table S3). At knee flexion of 60° and 90°, the internal rotation angle in Group B was increased compared with Group A, and it was decreased significantly in Group C (Supplementary Table S3), and finally, the internal rotation angle in Group A and C had no difference (Supplementary Table S3). The anterior tibial displacement between Group A, B and C had no significant difference, regardless the different knee flexion angle (Supplementary Table S4).

### Summary Between Groups

1) Comparisons between Group A and B: the internal rotation angle in Group B was increased significantly at knee flexion of 30°, 60° and 90° than Group A; the anterior tibia displacement in Group B was increased significantly at knee flexion of 30° and 60° than Group A; at knee valgus, the internal rotation angle in Group B was also increased significantly at knee flexion of 60° and 90°than Group A.
2) Comparisons between Group B and C: the internal rotation angle in Group C was decreased significantly at knee flexion of 30°, 60 than Group B; the anterior displacement in Group C was decreased significantly at knee flexion of 30° and 60° than Group B; at valgus, the internal rotation angle in Group C was also decreased significantly at knee flexion of 60° and 90° than Group B.
3) Comparisons between Group A and C: only the internal rotation angle and anterior displacement at knee flexion of 30° in Group C were increased than Group A.

## Discussion

LMPRT mainly affects the rotational stability of knee joint, even when ACL is intact. Both of the LMPR and ACL are the stabilizers during knee motion. With the increasing of knee flexion, the LMPR will replace the ACL, becoming the primary stabilizer for preventing the over-internal rotation and over-forward shift. (Pache et al., 2018). This biomechanical kinematics cadaver study found that the LMPRT can lead to notable internal rotational instability when knee flexion ranged from 30° to 90°, when ACL was tensioned by the valgus load in the test, the LMPRT can also lead to notable internal rotational instability when knee flexion ranged from 60° to 90°. The results indicated that the knee stability contributed by the LMPR can not be totally replaced by an intact ACL. Shybut et al. reported that LMPRT can further reduce the knee rotational stability. (Levy et al., 1982). What’s more, our results also found that the LMPRT repairing can completely restore the stability in most cases, and significantly improve the internal rotation and anterior shift stability at knee flexion of 30° compared with the LMPRT group.

LMPRT affects the anterior shift stability as well. This cadaver study found that the LMPRT can lead to notable shift stability instability when knee flexion ranged from 30° to 60°, and the LMPRT repairing can successfully restore the stability. Similar to our results, Tang et al. also found that LMPRT can reduce the knee stability after ACL reconstruction, while LMPRT repairing may restore the stability on tibial axial and anterior displacement. (Tang et al., 2019). Frank et al. evaluated the biomechanical effects of LMPRT the knee without ACL, and they found that the anterior tibial motion further increase significantly when knee flexion at 30°. (Frank et al., 2017).

We consider that it is very essential to repair the LMPRT, since the knee stability contributed by LMPR can not be totally replaced by intact ACL, for example, a reconstructed ACL. In fact, the ACL rupture combined with LMPRT is common in clinical. (Ahn et al., 2009; Ahn et al., 2010; Feucht et al., 2015). The researchers reported that in ACL reconstruction, the proportion of LMPR lesions is 7–12%. (You et al., 19872014; Forkel and Petersen, 2012). In a study based on MRI, Brody et al. found that in the case of ACL injury, LMPRT was more common than the medial meniscus. (Brody et al., 2006). There is still a controversy on the necessity of LMPRT repair, some scholars believe that the conservative treatments can restore the normal function of knee joint, rather than the operation, (Lim et al., 2010), while others prefer the LMPRT repair operation because of the intact meniscus function and better prognosis. (Allaire et al., 2008). The biomechanical and kinematic studies on how the LMPRT affecting the stability are necessary to make the final decision. In this study, we had proved that in LMPRT group, the knee stability can not be totally compensated by an intact ACL, especially the internal rotational instability at knee flexion, even when ACL is tensioned, which suggests that the LMPRT should be repaired together with the ACL reconstruction. Petrigliano et al. also found that LMPRT can still reduce the stability of lateral compartment during the axial shift test after the ACL anatomical reconstruction. (Petrigliano et al., 2011). Besides, the reconstructed ACL is not strong enough to provide sufficient rotational stability. Knee instability is a risk factor for the degeneration and cartilage injury, and LMPRT can also promote the process of knee osteoarthritis. Hence, we considered that LMPRT should be repaired together with ACL reconstruction. Jin et al. performed ACL reconstructions and all-inside sutures for LMPRT in 25 patients, after 18 months follow-up, all patients exhibited favorable clinical results, (Jin Hwan et al., 2010), which supports our results and conclusion.

The present study had several limitations. First, this was a static mechanical study, and we can not analyze the dynamic kinematics effects of the LMPRT on knee stability. Further biomechanical dynamic kinematics studies are needed to evaluated the effects of LMPRT on dynamic knee stability during motion. Second, the sample size of this experiment was relatively small, which may affect the accuracy of results. Third, this was a simulated mechanical study in cadaver, lacking of the muscular control, which can also affect the joint stability. Hence the results and conclusion need to be further confirmed by clinical case-control as well as longitudinal studies.

## Conclusion

LMPR is essential for the rotational stability and anterior shift stability at knee flexion. In simulated knee with intact ACL, LMPRT can still lead to the notable internal rotational instability when knee flexion ranged from 30° to 90°, as well as the anterior shift instability when knee flexion ranged from 30° to 60°, while LMPRT repair help to improve the internal rotation and anterior shift stability at 30° compared with the LMPRT group, and restore the internal rotation stability at 60° to 90°, as well as the anterior shift stability at 60°. Our study provided a biomechanical kinematics basis for the operation necessity of LMPRT repair.

## Data Availability

The original contributions presented in the study are included in the article/Supplementary Material, further inquiries can be directed to the corresponding authors.
