# Supplementary material for: The Effects of Lateral Meniscus Posterior Root Tear and its Repair on Knee Stability of Internal Rotation and Forward Shift: A Biomechanical Kinematics Cadaver Study
Source: Front Bioeng Biotechnol. 2022 Jan 19;9:792894. doi: 10.3389/fbioe.2021.792894 (PMC8807685; doi:10.3389/fbioe.2021.792894)
Supplement: Supplementary file 1 [file DataSheet3.docx]

A C D


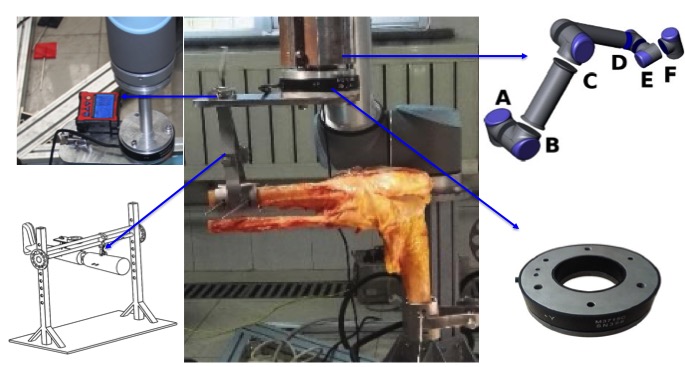


B E

Fig. 1 The biomechanical kinematics experiment test platform

Fig 1A, the digital angle measuring instrument; Fig 1B, a self-made corpse knee fixture; Fig 1D, the UR10 robotic arm; Fig 1E, 6-demesional freedom force sensors


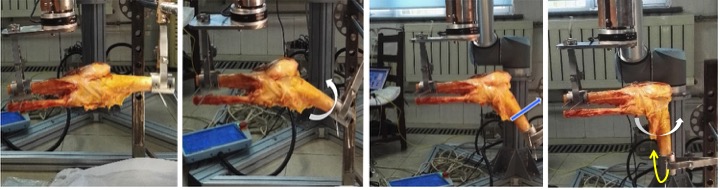
A B C D

Fig.2 Biomechanical kinematics parameters and procedures

Fig 2A, at the beginning, the knee was fixed with Kirschner wires and clamps at 0°; Fig 2B, during the internal rotation stability test, an internal rotation load of 5 N·m (while arrow) was given to tibia, this figure shows the internal rotation angle was detected at 30°; Fig 2C, during the anterior shift stability test, a forward shifting load of 134N (blue arrow) was given to the tibia, and this figure shows the anterior displacement was measured at 60°; Fig 2D, during stability test knee valgus, an internal rotation load of 5 N·m (while arrow) and a valgus stress with 10 N·m (yellow arrow) were given to the tibia, this figure shows the internal rotation angle and anterior displacement were detected at 90°.
