# Supplementary material for: The Effects of Lateral Meniscus Posterior Root Tear and its Repair on Knee Stability of Internal Rotation and Forward Shift: A Biomechanical Kinematics Cadaver Study
Source: Front Bioeng Biotechnol. 2022 Jan 19;9:792894. doi: 10.3389/fbioe.2021.792894 (PMC8807685; doi:10.3389/fbioe.2021.792894)
Supplement: Supplementary file 3 [file DataSheet1.docx]

**Table 1****. The tibia internal rotation angles by internal rotation moment of 5N**·m

| Internal rotation (°) | Knee flexion | | | |
| --- | --- | --- | --- | --- |
|  | 0 ° | 30 ° | 60 ° | 90 ° |
| Group A | 9.07 ± 0.72 | 16.01 ± 0.83 | 14.62 ±1.07 | 12.80 ±1.56 |
| Group B | 9.19 ± 0.54 | 17.88 ± 1.08* | 16.31 ±1.29* | 16.78 ±1.23* |
| Group C | 9.11 ± 0.63 | 16.84± 1.01* ^#^ | 14.33 ±1.10^#^ | 14.14 ±1.15^#^ |
| P_ANOVA_ | 0.821 | 0.019 | 0.036 | 0.015 |
| *P_A-B_* | 0.841 | 0.025 | 0.041 | 0.002 |
| *P_B-C_* | 0.955 | 0.030 | 0.019 | 0.021 |
| *P_A-C_* | 0.951 | 0.047 | 0.675 | 0.416 |

Note: comparisons were analyzed by one-way *ANOVA*, and *P* values between groups were performed by *LSD* method; Group A: the lateral MPR was intact; Group B: the lateral MPR was cut off from its tibial end; Group C: the LMPRT has been repaired; *, *P*<0.05, comparing to Group A; # , *P*<0.05, comparing to Group B.

**Table 2.** **The tibia anterior shift displacements by the forward force of 134N**

| Anterior shift (mm) | Knee flexion | | | |
| --- | --- | --- | --- | --- |
|  | 0 ° | 30 ° | 60 ° | 90 ° |
| Group A | 3.33 ±0.55 | 3.67± 0.73 | 3.89±0.42 | 3.75±0.70 |
| Group B | 3.56 ±0.49 | 5.01± 0.64* | 4.60±0.51* | 3.77±0.82 |
| Group C | 3.47 ±0.37 | 4.54± 0.82* ^#^ | 4.18±0.49^#^ | 3.99±0.64 |
| P_ANOVA_ | 0.420 | 0.003 | 0.043 | 0.773 |
| *P_A-B_* | 0.648 | 0.015 | 0.024 | 0.998 |
| *P_B-C_* | 0.450 | 0.042 | 0.037 | 0.816 |
| *P_A-C_* | 0.233 | 0.033 | 0.207 | 0.450 |

Note: comparisons were analyzed by one-way *ANOVA*, and *P* values between groups were performed by *LSD* method; Group A: the lateral MPR was intact; Group B: the lateral MPR was cut off from its tibial end; Group C: the LMPRT has been repaired; *, *P*<0.05, comparing to Group A; # , *P*<0.05, comparing to Group B.

**Table 3****. The tibia internal rotation angles by internal rotation moment of 5N**·m **and valgus moment of 10N**·m

| Internal rotation (°) | Knee flexion | | | |
| --- | --- | --- | --- | --- |
|  | 0 ° | 30 ° | 60 ° | 90 ° |
| Group A | 9.26 ± 0.92 | 14.36±3.21 | 14.89±1.47 | 14.37±0.58 |
| Group B | 9.31 ± 1.84 | 14.41±1.38 | 17.13±0.88* | 16.52±1.07* |
| Group C | 9.29 ± 1.22 | 15.02±1.77 | 14.68±1.10 ^#^ | 14.38±2.04 ^#^ |
| P_ANOVA_ | 0.965 | 0.731 | 0.013 | 0.042 |
| *P_A-B_* | 0.935 | 0.878 | 0.011 | 0.037 |
| *P_B-C_* | 0.951 | 0.331 | 0.013 | 0.045 |
| *P_A-C_* | 0.998 | 0.132 | 0.650 | 0.877 |

Note: comparisons were analyzed by one-way *ANOVA*, and *P* values between groups were performed by *LSD* method; Group A: the lateral MPR was intact; Group B: the lateral MPR was cut off from its tibial end; Group C: the LMPRT has been repaired; *, *P*<0.05, comparing to Group A; # , *P*<0.05, comparing to Group B.

**Table 4. The tibia anterior shift displacements by internal rotation moment of 5N**·m **and valgus moment of 10N**·m

| Anterior shift (mm) | Knee flexion | | | |
| --- | --- | --- | --- | --- |
|  | 0 ° | 30 ° | 60 ° | 90 ° |
| Group A | 0.72 ±0.17 | 1.87 ±0.14 | 0.45±0.18 | 0.23 ±0.11 |
| Group B | 0.71 ±0.21 | 1.73 ±0.17 | 0.52±0.09 | 0.25 ±0.07 |
| Group C | 0.70±0.19 | 1.85 ±0.16 | 0.48±0.20 | 0.20 ±0.09 |
| P_ANOVA_ | 0.907 | 0.688 | 0.787 | 0.552 |
| *P_A-B_* | 0.911 | 0.561 | 0.230 | 0.693 |
| *P_B-C_* | 0.989 | 0.346 | 0.798 | 0.378 |
| *P_A-C_* | 0.901 | 0.833 | 0.817 | 0.450 |

Note: comparisons were analyzed by one-way *ANOVA*, and *P* values between groups were performed by *LSD* method; Group A: the lateral MPR was intact; Group B: the lateral MPR was cut off from its tibial end; Group C: the LMPRT has been repaired; *, *P*<0.05, comparing to Group A; # , *P*<0.05, comparing to Group B.
